# Structural and molecular heterogeneity of calretinin‐expressing interneurons in the rodent and primate striatum

**DOI:** 10.1002/cne.24373

**Published:** 2017-12-21

**Authors:** Farid N. Garas, Eszter Kormann, Rahul S. Shah, Federica Vinciati, Yoland Smith, Peter J. Magill, Andrew Sharott

**Affiliations:** ^1^ Medical Research Council Brain Network Dynamics Unit, Department of Pharmacology University of Oxford Oxford United Kingdom; ^2^ Yerkes National Primate Research Center, Department of Neurology and Udall Center of Excellence for Parkinson's Disease Research Emory University Atlanta Georgia

**Keywords:** calretinin, interneuron, neuronal diversity, RRID: AB_10000342, RRID: AB_94259, RRID: AB_2228331, RRID: AB_2269934, RRID: AB_10864618, RRID: AB_2194626, RRID: AB_2064130, secretagogin, striatum

## Abstract

Calretinin‐expressing (CR+) interneurons are the most common type of striatal interneuron in primates. However, because CR+ interneurons are relatively scarce in rodent striatum, little is known about their molecular and other properties, and they are typically excluded from models of striatal circuitry. Moreover, CR+ interneurons are often treated in models as a single homogenous population, despite previous descriptions of their heterogeneous structures and spatial distributions in rodents and primates. Here, we demonstrate that, in rodents, the combinatorial expression of secretagogin (Scgn), specificity protein 8 (SP8) and/or LIM homeobox protein 7 (Lhx7) separates striatal CR+ interneurons into three structurally and topographically distinct cell populations. The CR+/Scgn+/SP8+/Lhx7− interneurons are small‐sized (typically 7–11 µm in somatic diameter), possess tortuous, partially spiny dendrites, and are rostrally biased in their positioning within striatum. The CR+/Scgn−/SP8−/Lhx7− interneurons are medium‐sized (typically 12–15 µm), have bipolar dendrites, and are homogenously distributed throughout striatum. The CR+/Scgn−/SP8−/Lhx7+ interneurons are relatively large‐sized (typically 12–20 µm), and have thick, infrequently branching dendrites. Furthermore, we provide the first in vivo electrophysiological recordings of identified CR+ interneurons, all of which were the CR+/Scgn−/SP8−/Lhx7− cell type. In the primate striatum, Scgn co‐expression also identified a topographically distinct CR+ interneuron population with a rostral bias similar to that seen in both rats and mice. Taken together, these results suggest that striatal CR+ interneurons comprise at least three molecularly, structurally, and topographically distinct cell populations in rodents. These properties are partially conserved in primates, in which the relative abundance of CR+ interneurons suggests that they play a critical role in striatal microcircuits.

## 1 INTRODUCTION

The striatum is the major site of extrinsic inputs to the basal ganglia and mostly consists of GABAergic spiny projection neurons (SPNs) that target other basal ganglia nuclei. The output of SPNs is thought to be sculpted by an array of different types of striatal interneurons. GABAergic interneurons expressing the calcium‐binding protein (CBP) calretinin (CR) are the most abundant class of interneuron in the primate striatum (Cicchetti, Prensa, Wu, & Parent, 2000; Wu & Parent, 2000), and recent evidence suggests that newborn calretinin‐expressing (CR+) neurons continue to be added to the adult striatum (Ernst et al., 2014; Wei et al., 2011). Despite this, CR+ interneurons remain the least well understood of the four “classical” striatal interneuron types, and their in vivo firing properties have never been characterized (Silberberg & Bolam, 2015; Tepper, Tecuapetla, Koos, & Ibanez‐Sandoval, 2010). CR+ interneurons in the neocortex (Miettinen, Gulyas, Baimbridge, Jacobowitz, & Freund, 1992) are comprised of multiple cell types with distinct structural, molecular and physiological features (Ascoli et al., 2008; Klausberger & Somogyi, 2008), and several lines of evidence suggest that this may also be the case in striatum.

In both mice and rats, striatal CR+ interneurons can be separated into at least two groups based on cell body size (Petryszyn, Beaulieu, Parent, & Parent, 2014; Rymar, Sasseville, Luk, & Sadikot, 2004). In the mouse, small‐sized CR+ interneurons are more common in the rostral and dorsal parts of striatum (Petryszyn et al., 2014). Many CR+ interneurons in these areas of rodent striatum express the transcription factor SP8 (Wei et al., 2011) or the CBP secretagogin (Kosaka, Yasuda, & Kosaka, 2017), but whether the expression of these molecules correlates with soma size in these areas has not been investigated. In the monkey striatum, three populations of CR+ interneurons have also been described based on their structural properties (Parent, Fortin, Cote, & Cicchetti, 1996; Petryszyn et al., 2014). In order of decreasing prevalence, there is a group comprised of “medium‐sized” (10–20 μm in somatic diameter) CR+ cells (Wu & Parent, 2000), a group of “small‐sized” (10–12 μm) cells, and a group of “large‐sized” (25–40 μm) cells. Although there is no evidence of molecular divergence of small‐ and medium‐sized interneurons in primates, the large‐sized CR+ interneurons often co‐express choline acetyltransferase (ChAT) (Cicchetti, Beach, & Parent, 1998; Petryszyn et al., 2014). This represents an important difference between the primate and rodent striatum, as neither mice nor rats have interneurons co‐expressing ChAT and CR (Figueredo‐Cardenas, Medina, & Reiner, 1996; Petryszyn et al., 2014). Without this molecular correlate of large CR+ interneurons, it is unclear whether the rodent striatum has two (Petryszyn, Parent, & Parent, 2017) or three (Tepper et al., 2010) classes of CR+ interneuron.

In both rodents and primates, a more detailed quantification of the molecular identity of CR+ neurons with different structural properties is needed to better differentiate subtypes of CR+ interneuron. Such a characterization could also provide greater understanding of the role of these interneurons in patients with Huntington's disease and Tourette syndrome, where there is a preferential loss of large‐sized striatal CR+ interneurons and relative sparing of the medium‐sized cells (Cicchetti et al., 2000; Kataoka et al., 2010). Using a combination of immunohistochemistry and stereological cell counting, we demonstrate that the selective expression of Scgn, as well as the transcription factors SP8 and Lhx7, within the CR+ interneuron population can be used to identify three cell “types” that can be distinguished from one another on the basis of their structural properties and distribution within the dorsal striatum of the rat and mouse. We also demonstrate that one of these markers, Scgn, also identifies a subpopulation of CR‐expressing interneurons that is unevenly distributed throughout the caudate‐putamen in primates. Together these results provide further evidence of functionally distinct subpopulations of CR+ interneurons in the rodent and primate striatum.

## 2 MATERIALS AND METHODS

### 2.1 Preparation of rat and mouse brain tissue for immunofluorescence and cell counting

The experimental procedures described below were carried out using 14 adult (3 months old, 280–350 g) male Sprague Dawley rats (Charles River) and 6 adult, 3‐month‐old C57Bl/6J male mice (Charles River) in accordance with the Animals (Scientific Procedures) Act, 1986 (UK). After being deeply anaesthetized using isoflurane (4% v/v in oxygen), each rat was given a lethal dose of pentobarbitone (1.3 g/kg; i.p.) followed by transcardial perfusion with approximately 50 mL of 0.05 M phosphate‐buffered saline, pH 7.4 (PBS), followed by 300 mL of fixative (4% w/v paraformaldehyde with 0.1% w/v glutaraldehyde in 0.1 M phosphate buffer, pH7.4 (PB)). This was followed by a third perfusion of approximately 200 mL of fixative (4% w/v paraformaldehyde in PB). Mice were deeply anesthetized with pentobarbitone and perfused transcardially using 20 mL of PBS, followed by 20 mL of fixative (4% w/v paraformaldehyde in 0.1 M PB). For both species, once the brain was removed, the tissue was post‐fixed in this solution for 24 hr at 4°C. Using a vibrating microtome (Leica VT1000S), 50‐µm‐thick coronal sections containing the striatum were cut and collected for immunofluorescence processing. For some images, 50‐µm‐thick parasagittal sections were used to visualize the medial striatum.

### 2.2 Indirect immunofluorescence

Sections were washed with PBS and pre‐incubated for 2 hr in a solution consisting of 10% v/v NDS (normal donkey serum) and 0.3% v/v Triton X‐100 in PBS. After further washing using PBS, sections were incubated overnight at room temperature in a solution of 0.3% v/v Triton X‐100 in PBS containing primary antibodies (RRID: AB_10000342, RRID: AB_94259, RRID: AB_2228331, RRID: AB_2269934, RRID: AB_10864618, RRID: AB_2194626, RRID: AB_2064130; see Table 1 for details about the sources and dilutions of antibodies used). After exposure to primary antibodies, sections were washed in PBS and incubated overnight at room temperature in Triton‐PBS which contained a mixture of secondary antibodies (all raised in donkey) which were conjugated to the following fluorophores: DyLight 649 (1:500; Jackson ImmunoResearch Laboratories); Cy3 (1:1,000; Jackson ImmunoResearch Laboratories); AlexaFluor‐488 (1:500; Invitrogen); or AMCA (1:250 dilution; Jackson ImmunoResearch Laboratories). To ensure minimal cross‐reactivity, these antibodies were cross adsorbed by the manufacturers. After further washing in PBS, sections were mounted on glass slides (VWR Super Premium Microscope slides) using fluorescence mounting medium (Vectashield; Vector Laboratories), followed by the addition of a coverslip.

**Table 1:** Primary antibodies used in this study

Molecular marker	Host Animal	Dilution	Source and catalog number	B‐cell lineage	Research Resource Identifier (RRID)	Specificity reference
Calretinin (CR)	Goat	1:500	Swant CG1	Polyclonal	AB_10000342	Simmons, Tong, Schrader, and Hornak (2010)
Calretinin (CR)	Mouse	1:500	Millipore MAB1568	Monoclonal	AB_94259	Ingham, Gunhan, Fuller, and Fuller (2009)
Calretinin (CR)	Rabbit	1:1,000	Synaptic Systems 214102	Polyclonal	AB_2228331	
LIM‐homeobox 7/8 (Lhx7)	Guinea Pig	1:500	Gift, A. Rajkovic	Polyclonal	N/A	Pangas et al. (2006)
Secretagogin (Scgn)	Goat	1:1,000	R&D systems AF4878	Polyclonal	AB_2269934	See below^1^
Secretagogin (Scgn)	Rabbit	1:500	Abcam AB111871	Polyclonal	AB_10864618	See below^1^
Specificity protein 8 (SP8)	Goat	1:30,000	Santa Cruz SC‐104661	Polyclonal	AB_2194626	Cai et al. (2013)
COUP TF1‐interacting protein 2 (Ctip2)	Rat	1:500	Abcam AB18465	Monoclonal	AB_2064130	Huang, Yu, Shimoda, Watanabe, and Liu (2012)
Choline acetyltransferase (ChAT)	Mouse	1:250	Gift, C. Cozzari mAb5	Monoclonal	N/A	von Engelhardt, Eliava, Meyer, Rozov, and Monyer (2007)

1 – Goat anti‐Scgn and rabbit anti‐Scgn were raised against different secretagogin peptide antigens but resulted in a significant overlap in immunoreactive neurons (Garas et al., 2016).

### 2.3 Sampling and cell‐counting strategies

Using a series of partly overlapping, complementary immunofluorescence protocols, striatal neurons were tested for their combinatorial expression of the molecular markers in Table 1. A version of design‐based stereology, the “modified optical fractionator” (Abdi et al., 2015; West, 1999), was used to generate unbiased cell counts, determine the relative expression of molecular markers, and map distributions of striatal interneurons. In all procedures performed, the accuracy of these estimates was ensured by taking absolute counts of all neurons expressing a given molecular marker, thereby allowing for a nearly precise definition of their distribution within the striatum.

For analyses of rat striatum, the expression of specific molecular markers was quantified using 13 coronal striatal planes that traversed almost the entire extent of the dorsal striatum (coordinates along the rostral–caudal axis: 2.3, 1.8, 1.2, 0.6, −0.1, −0.5, −0.9, −1.3, −1.8, −2.2, −2.6, −3.0, and −3.3 mm from Bregma). For analyses of mouse dorsal striatum, 9 coronal planes were used (coordinates: 1.7, 1.3, 0.9, 0.5, 0.2, −0.1, −0.5, −1.0, and −1.5 mm from Bregma). Sections from different animals of the same species were matched to similar stereotaxic planes along the rostral‐caudal axis (±50 µm) using the appropriate atlas (Franklin & Paxinos, 2008; Paxinos & Watson, 2007).

Once the chosen striatal coronal planes were identified and incubated in primary and secondary antibodies, an epifluorescence microscope (Carl Zeiss, AxioImager.M2) running Axiovision software (Carl Zeiss) and equipped with a 20X (Numerical Aperture = 1.8) objective and StereoInvestigator 9.0 software (MBF Biosciences) was used to delineate the dorsal striatum. In order to image each fluorescence channel, the following sets of filter cubes were used: AMCA (excitation 299–392 nm, beamsplitter 395 nm, emission 420–470 nm); AlexaFluor‐488 (excitation 450–490 nm, beamsplitter 495 nm, emission 500–550 nm); Cy3 (excitation 532–558 nm, beamsplitter 570 nm, emission 570–640 nm); and DyLight 649 (excitation 625–655 nm, beamsplitter 660 nm, emission 665–715 nm). The borders of the dorsal and ventral striatum were determined using the boundaries defined in standard stereotaxic atlases for rat (Paxinos & Watson, 2007) and mouse (Franklin & Paxinos, 2008).

Once boundaries were delineated for a given section that had undergone a given immunofluorescence protocol, the selected area was subsequently captured by imaging a series of completely tessellated, z‐stacked images (with an optical step size of 1 µm) at depths 2–12 μm from the upper surface of each section at the level of the striatum. This was done using the 20× 1.8 NA objective lens of an epifluorescence microscope (as above). The thickness of the section was measured at each counting site and then averaged to obtain a correction factor for tissue shrinkage. The average section thickness was found to be 47.8 ± 0.73 µm in rats meaning that the calculated shrinkage factor was ∼4.4%. In mice, the average section thickness was found to be 46.5 ± 0.66 µm, resulting in a calculated shrinkage factor of ∼7.0%. To minimize confounds arising from surface irregularities, neuropil within a 2 μm “guard zone” at the upper surface was not imaged. By sampling sections in this manner, a 10 µm‐thick “optical disector” was generated which had abutting, unbiased 2D counting frames (320 × 420 µm) consisting of two perpendicular exclusion lines and two inclusion lines. This was used to generate all stereological cell counts and molecular expression profiles presented in this study (Garas et al., 2016; Glaser, Greene, & Hendricks, 2007; West, 1999, 2012), and allowed for the generation of robust and unbiased stereological cell counts.

Captured images were analyzed and labeled neurons were counted using StereoInvestigator 9.0 software (MBF Biosciences). A labeled neuron was counted if the top of its nucleus came into focus within the optical disector. If the nucleus was already in focus at the top of the optical disector, the neuron was excluded (West, 1999). As strongly fluorescent cytoplasmic/nuclear markers can obscure nuclear boundaries, delineation of these nuclei was achieved when necessary by incubating the tissue for 10 min in a solution (1:100,000) of the DNA dye 4′,6‐diamidino‐2‐phenylindole (DAPI). DAPI was used solely to define whether or not the nucleus of a given cell was within the optical disector and thus, DAPI signals were not included in any stereological counts nor used to define somatic diameters. For a given molecular marker, X, positive immunoreactivity (confirmed expression) was denoted as X+, whereas undetectable immunoreactivity (no expression) was denoted as X−. In all fluorescence analysis, a neuron was classified as not expressing the tested marker only when positive immunoreactivity could be observed in other cells on the same optical section as the tested neuron. Each immunofluorescence protocol described in this study was repeated in a minimum of three adult rats/mice. The data obtained from each count was subsequently exported from StereoInvestigator (MBF Biosciences) into Excel (Microsoft) or MATLAB (Mathworks), where they were pooled for further analysis.

### 2.4 Calculations of section area, volume and estimates of total cell number

Once the number of neurons expressing a marker or markers had been counted and recorded for a given coronal plane, the volume of the striatum in which these neurons had been counted was calculated using StereoInvestigator 9.0 software (MBF Biosciences). The software uses a method that is derivative of the point counting method (Oorschot, 1996). The volume of tissue within the optical disector was calculated by multiplying the cross‐sectional area by 10 µm. The density of labeled neurons was then computed by dividing the total number of counted neurons by the volume of the optical disector. By assuming that the density of immunoreactive neurons is constant within the 50‐µm‐thick tissue section (corrected for tissue shrinkage in mice and rats), then the calculated value for absolute density applies to the entire section. This absolute density value was calculated for every coronal plane studied for all counted neurons, and the values were plotted to demonstrate changes in density along the rostro‐caudal axis of the dorsal striatum. A calculation for an estimate of the mean density of counted neurons within the striatum was performed by computing the mean of all densities for each coronal plane that was studied.

In order to calculate an estimate for the total number of a given type of neuron in the striatum, the following equation (Oorschot, 1996) was used:

N= Nv ∗V(ref),

where *N* is the estimate for the total number of neurons in the structure, Nv is the numerical or volume density, and *V*(ref) is the volume of that structure.

The volume of the dorsal striatum in rats and mice was calculated using the Cavalieri direct volume estimate (Gundersen & Jensen, 1987), which involves multiplying the sum of the cross‐sectional area of every nth serial section multiplied by the fixed distance between each of the sampled sections. The volume of the striatum in both rats and mice was calculated using 13 and 9 equally spaced 50‐µm‐thick coronal sections, respectively (Gundersen & Jensen, 1987; Rymar et al., 2004). The point counting method option of the StereoInvestigator 9.0 software was used to obtain an estimate of each of the section's cross‐sectional area. Briefly, this involved overlaying a grid of equally spaced points over each delineation of the structure, and then counting the number of points that lie within the contour of the structure (West, 1999). The spacing of the points is often set such that approximately 150 points lie within the entire of set of contours for a given structure. This allows for an accurate measurement of each section's area.

Once complete, the volume of the structure, *V*(ref), was calculated according to the following (Oorschot, 1996; West, 1999):

V(ref) = ∑P∗a(p) ∗t

where *P* is the number of points counted within a delineated contour, *a*(p) is the area represented by each point, and *t* is the fixed distance between each section. When calculated in this manner across the striatum of six rats, the mean volume of the dorsal striatum was determined to be 24.2 ± 2.1 mm^3^, a value that resembles previous estimates (Oorschot, 1996; Rymar et al., 2004). The mean volume of the mouse striatum (*n* = 6 mice) was determined to be 9.4 ± 0.5 mm^3^. Once the value of *V*(ref) is determined, multiplying that volume by the mean neuronal density yields an estimate for the total number of neurons for that animal. Because most neuronal counts were performed across three animals (a sample that is typical of stereological studies), in depth statistics were deemed inappropriate, and so the value calculated from each animal is plotted along with the mean.

In order to estimate the precision of this estimate for the volume of striatum, the coefficient of error (CE) was calculated using the Stereo investigator software. In all such studies, a CE (*m* = 1) less than 0.1 is the agreed upon value which indicates that the level of variation in striatal volume calculation between different animals is a product of biological, rather than methodological variation (Gundersen, Jensen, Kieu, & Nielsen, 1999). In this study, the coefficient of error was kept below the value of 0.1 for each animal by first ensuring that each section's cross sectional area was calculated using approximately 150 points as per the point counting method. Secondly, because each volume estimate was performed using 13 striatal sections in the rat and 9 striatal sections in the mouse, this ensured that the striatum cross‐sectional area was sampled at approximately every 9th section in the rat and every 7th section in the mouse. This degree of sampling has previously been shown to ensure a CE of less than 0.1 when estimating the volume of complex structures (Schmitz & Hof, 2005; Slomianka & West, 2005).

### 2.5 Topographical and statistical analysis of interneuron distribution

The positions of individual interneurons and the volume of striatum calculated by StereoInvestigator for each section were imported into MATLAB (Mathworks, version: R2014b). Using previously described analyses (Garas et al., 2016), we then performed quantitative analyses of the spatial distribution of each cell type. Briefly, for all counted neurons in the dorsal striatum, horizontal, and vertical lines were first placed from the position of each interneuron within a given coronal plane. Each interneuron was then assigned a value of between −1 and 1 according to its relative distance from the medio‐lateral and dorso‐ventral borders of striatum at that same coronal plane. Notably, these two distance values were calculated according to the first points along the striatal contours that were contacted by the horizontal and vertical lines drawn from each neurons position. In this manner, the definition of “medial” and “lateral” striatum was shifted depending on the position of the interneuron in the dorso‐ventral axis and vice versa. This method of normalization therefore accommodates the irregular, ever changing shape of the dorsal striatum when viewed over multiple coronal planes.

As each counted interneuron in a section was given a value for each axis, and at least 3–6 animals were used for each protocol, this sample size was deemed large enough to use the Wilcoxon signed‐rank test in order to determine if the mean relative value of a neural population in a given coronal plane differed significantly from 0, a value indicating an unbiased distribution along a given axis. For coronal planes in which 5 or fewer neurons of a given class were counted within the confines of the optical disector, the sample size was deemed too small for the generation of reproducible statistics. The minimum significance level for these statistical tests was taken to be *p* ≤ .05, and is denoted in plots by the presence of an asterisk (*). Mann‐Whitney *U* tests where used to compare the mean positional values of two different interneuron populations along the medio‐lateral or dorso‐ventral axis within a given coronal plane. The minimum significance level for these statistical tests was taken to be *p* ≤ .05, and is denoted by the presence of a box (□). Multiple comparisons across sections were corrected using the false discovery rate (FDR) method (Noble, 2009).

### 2.6 Measurements of mean somatic diameters of interneurons

Immunofluorescent detection of markers that labeled the entirety of the somata of certain classes of CR+ interneurons was used in order to measure the somatic diameters of individual neurons in the dorsal striatum of the rat, mouse and primate. A neuron was only measured if the contours of its entire soma in all three dimensions were contained within the 50‐µm‐thick section of tissue. As previously described (Rymar et al., 2004), this was done in order to ensure that the longest axis of a chosen neuron's soma was imaged. The somatic diameter of a labeled neuron was defined as the length of its longest axis, and was measured using StereoInvestigator software. For a given class of interneurons, the mean of all somatic diameters measured in this manner was calculated. Statistical comparisons of multiple mean somatic diameters (of multiple classes of interneuron) were performed using a Kruskal‐Wallis ANOVA followed by post hoc Dunn tests (where appropriate).

### 2.7 Imaging of immunolabeled CR‐expressing neurons

As shown previously, the (partial) somatodendritic features of some CR+ neurons can be visualized simply using immunohistochemistry (Bennett & Bolam, 1993; Petryszyn et al., 2017; Tepper et al., 2010). As such, the 40× lens (NA: 0.8) of a laser scanning confocal microscope (Zeiss LSM 710) was used to image well‐labeled CR‐expressing interneurons at multiple optical planes in order to form a z‐stack to highlight their somatodendritic structure. Single‐plane images highlighting the somata of these neurons were also taken in order to determine whether these neurons co‐expressed combinations of secretagogin, SP8, and Lhx7. In order to illustrate the rostral bias of those CR+ interneurons that co‐expressed SP8, parasagittal sections containing the medial striatum, the lateral ventricle and the subventricular zone were co‐labeled for CR and SP8. Imaging of these sections was performed using the 5× (NA: 0.16) lens of a laser scanning confocal microscope (Zeiss LSM 710) in which the shutter was left completely open (Nakamura, Sharott, & Magill, 2014).

### 2.8 In vivo electrophysiological recording and juxtacellular labeling of individual interneurons

Juxtacellular recording and labeling of neurons was performed in four anesthetized male Sprague Dawley rats (280–350 g) in accordance with the Animals (Scientific Procedures) Act 1986 (UK). As described previously (Garas et al., 2016; Sharott, Doig, Mallet, & Magill, 2012), induction of general anesthesia was achieved using 4% v/v isoflurane in O_2_, and maintained with urethane (1.3 g/kg, i.p; ethyl carbamate; Sigma) followed by supplemental doses of ketamine (30 mg/kg, i.p.; Willows Francis) and xylazine (3 mg/kg i.p.; Bayer). Local anesthetic was used to infiltrate the wound margins (0.5% w/v bupivacaine; Astra). A stereotaxic frame (Kopf) was used to fix the animal in place, and a homeothermic heating device (Harvard Apparatus) was used to maintain the animal's core body temperature at 37 ± 0.5°C. Recording of the electrocorticogram (ECoG) was performed directly above the frontal (somatic sensory‐motor) cortex (4.0 mm rostral and 2.0 mm lateral of Bregma) (Paxinos & Watson, 2007), with the reference screw being placed above the ipsilateral cerebellum (Abdi et al., 2015; Sharott et al., 2012). The raw ECoG signal then underwent bandpass filtering (0.3–1,500 Hz, −3 dB limits) and amplification (2000×; DPA‐2FS filter/amplifier; NPI Electronic Instruments) prior to its acquisition. Using a computer‐controlled stepper motor (IVM‐1000; Scientifica), standard‐wall borosilicate glass electrodes (10–30 MΩ in situ with a tip diameter of approximately 1.5 μm) filled with a 0.5 M solution of NaCl containing neurobiotin (1.5% w/v; Vector Laboratories) were lowered into the dorsal striatum (under stereotaxic guidance). The depth of the electrode was recorded by the computer at a resolution of 0.1 μm. Action potentials of individual striatal neurons (i.e., single‐unit activity) were subsequently recorded after being amplified (10×) (Axoprobe‐1A amplifier; Molecular Devices), AC‐coupled, amplified a further 100×, and bandpass filtered at 300–5,000 Hz (DPA‐2FS filter/amplifier). Both the ECoG and single‐unit activity were recorded at a sampling rate of 16.6 kHz using a Power1401 Analog‐Digital converter and a PC running Spike2 acquisition and analysis software (Version 7.2; Cambridge Electronic Design). As described previously, brain state was defined based on the oscillatory content of the ECoG and categorized as either slow‐wave activity (SWA) or cortical activation (Garas et al., 2016; Magill, Bolam, & Bevan, 2000; Magill, Sharott, Bolam, & Brown, 2004; Sharott et al., 2012)

Once a neuron was recorded, it was juxtacellularly labeled with neurobiotin (Garas et al., 2016; Sharott et al., 2012). Such labeling was achieved by sending positive current pulses (2–10 nA, 200 ms, 50% duty cycle) through the electrode until the recorded single‐unit activity was “entrained” by the injection of current. Approximately 2–6 hr after labeling, animals were given a lethal dose of ketamine (150 mg/kg) followed by a transcardial perfusion with PBS, followed by fixative, as described above.

### 2.9 Tissue processing for identification of recorded and juxtacellularly labeled interneurons

Using a vibrating microtome (VT1000S; Leica Microsystems), 50‐μm‐thick coronal sections were serially collected and subsequently washed in PBS for 10 min. Sections were then incubated for a minimum of 4 hr in a solution of Triton PBS and Cy3‐conjugated streptavidin (1:1,000; Life Technologies). Sections were analyzed for Cy3 signal, and those containing neurobiotin‐labeled cells were isolated for further molecular characterization by indirect immunofluorescence (Garas et al., 2016; Sharott et al., 2012). Neurobiotin‐labeled neurons appearing to possess dendritic spines were defined as SPNs. In certain cases, they were also tested for their expression of Ctip2, a nuclear marker that has been shown to be expressed in the SPNs, but not in interneurons, in rodents. Neurons that did not express Ctip2, or had aspiny dendrites, were tested for their expression of one or more of the classical markers of striatal interneurons: CR, parvalbumin, choline acetyltransferase, and nitric oxide synthase. The initial molecular marker tested was guided by the labeled cell's somatodendritic structure and position within the striatum. Once a positive expression of one of these markers was established, no other classical marker was tested since these molecules are rarely co‐expressed in rodent striatal interneurons (Kawaguchi, 1993). Interneurons that expressed CR were further tested for their expression of secretagogin (Garas et al., 2016; Mulder et al., 2009) and Lhx7 (Pangas et al., 2006). All indirect immunofluorescence and subsequent epifluorescence and/or confocal imaging of neurobiotin‐labeled interneurons was performed using protocols described above for stereological cell counting and structural imaging of different subtypes of cells.

### 2.10 Data selection and analysis regarding the firing rate and regularity of identified striatal interneurons

Electrophysiological data were visually inspected, and epochs of robust cortical SWA or cortical activation were selected based on previous descriptions of these brain states (Garas et al., 2016; Sharott et al., 2012; Sharott, Vinciati, Nakamura, & Magill, 2017). Recordings were included for further analysis only when they were artifact free, and had a minimum duration of 50 s regardless of the brain state (185 ± 37.4 s; range 53–404 s). Spike sorting procedures such as template matching, principal component analysis and clustering (Mallet et al., 2008) were applied using Spike 2 software in order to isolate single‐unit activity. Isolation of a single unit was verified by the presence of a distinct refractory period in the interspike interval (ISI) histogram. Further analysis was later conducted after having converted single‐unit activity into a binary digital event (Spike 2) which was subsequently imported and analyzed using MATLAB software (Mathworks, version R2014b). Due to the low probability of “detecting” CR+ neurons using “blinded” in vivo recordings (see Results), only 4 CR+ interneurons were recorded and successfully labeled in the manner described above. As such, the average spike waveforms, firing rates and ISIs of all these recorded interneurons are individually presented.

### 2.11 Tissue preparation and indirect immunofluorescence in the caudate and putamen of the rhesus macaque

Monkey tissue used for stereological cell counting was obtained from two female adult rhesus monkeys (*Macaca mulatta*, 4.5–8.5 kg) from the Yerkes National Primate Research Center colony. All procedures performed were in accordance with guidelines from the National Institute of Health and were approved by Emory's Animal Care and Use Committee. The highly experienced Yerkes veterinary staff took care of the animals. The Yerkes National Primate Research Center is an NIH‐funded institution that is fully accredited by AAALAC, and regularly inspected by USDA. All activities at the Center are in compliance with federal guidelines. All experimental protocols concerning primates are performed in strict accordance with the NIH Guide for the Care and Use of Laboratory Animals and the PHS Policy on the Humane Care and Use of Laboratory Animals, and are reviewed and approved by the Emory IACUC before the proposed studies begin. Both monkeys were anesthetized using an intravenous injection of pentobarbital (100 mg/kg). This was followed by a transcardial perfusion with cold oxygenated Ringer's solution, and 2 L of a fixative solution containing 4% paraformaldehyde and 0.1% glutaraldehyde in PB (0.1 M, pH 7.4). Each brain was then cut into 10‐mm‐thick blocks in the coronal plane, which were subsequently cut into 50‐µm‐thick sections using a vibrating‐blade microtome. Sections containing the caudate/putamen were collected for use in immunofluorescence and cell counting procedures.

Immunofluorescence protocols were performed using 7 coronal sections which extended from the head of the caudate nucleus to the beginning of the tail of the caudate nucleus (coordinates along the rostro‐caudal axis: 4.1, 2.2, 0, −2.7, −5.2, −7.6, and −9.4 mm from Bregma). The sections from each monkey were matched such that the overall position of each section relative to Bregma (Paxinos, Huang, & Toga, 2000) from one monkey was similar to that in the second animal. These sections were washed with PBS and pre‐incubated for 2 hr in a solution consisting of 10% v/v NDS (normal donkey serum) and 0.3% Triton‐PBS.

Sections were incubated over three nights at room temperature in a solution of Triton‐PBS which contained primary antibodies against Scgn and CR. Table 1 contains the details, source and the dilutions used for these antibodies, as they did not differ from those used for rat and mouse tissue. The tissue was then washed in PBS before being incubated overnight at room temperature in a solution of Triton‐PBS and one of the fluorophore‐conjugated secondary antibodies. After further washing in PBS, sections were mounted on glass slides (VWR Super Premium Microscope slides) using fluorescence mounting medium (Vectashield; Vector Laboratories), followed by the addition of a coverslip. The caudate and putamen of each section were imaged using a Zeiss Imager M2 epifluorescence microscope equipped with a 20× objective and StereoInvestigator 9.0 software. The delineation of each structure was defined using the monkey brain atlas (Paxinos et al., 2000) and was done on a section‐by‐section basis. Once delineated, the acquisition and subsequent counting of labeled neurons was carried out in precisely the same manner as was done in rodent tissue (see above).

### 2.12 Analysis of stereological cell counts in the rhesus macaque

For each combination of immunofluorescence markers studied, the number of labeled neurons was counted in the caudate or the putamen (treated as separate structures) and recorded for a given coronal plane using the same principles described for the rodent. The calculation of *V*(ref) (i.e., an estimate of the volume of the caudate nucleus and the putamen) could not be accurately performed due to the lack of rhesus tissue containing the tail of the caudate nucleus (approximately 10 mm of caudate was not acquired). As a result, this study could not accurately report estimates for the total numbers of neurons in the entirety of the caudate nucleus or the putamen. As such, the absolute density for each counted neuron is presented rather than the total number, and was calculated by averaging the density of counted neurons within each coronal plane (as in the rat and mouse). This was done so that the results obtained from counts of different neuronal populations in different species performed in this study could be compared with one another.

The distribution of all counted neurons in the caudate nucleus and the putamen was analyzed and presented along the rostro‐caudal, dorso‐ventral, and medio‐lateral planes in the same manner as for the dorsal striatum of rodents. Statistical analysis regarding the distributional bias of counted neurons was also performed similarly to what was done for the results obtained in rodent dorsal striatum.

## 3 RESULTS

### 3.1 Topographically discrete populations of CR+ interneurons in the rat dorsal striatum can be identified by the selective expression of Scgn

**Figure 1:**
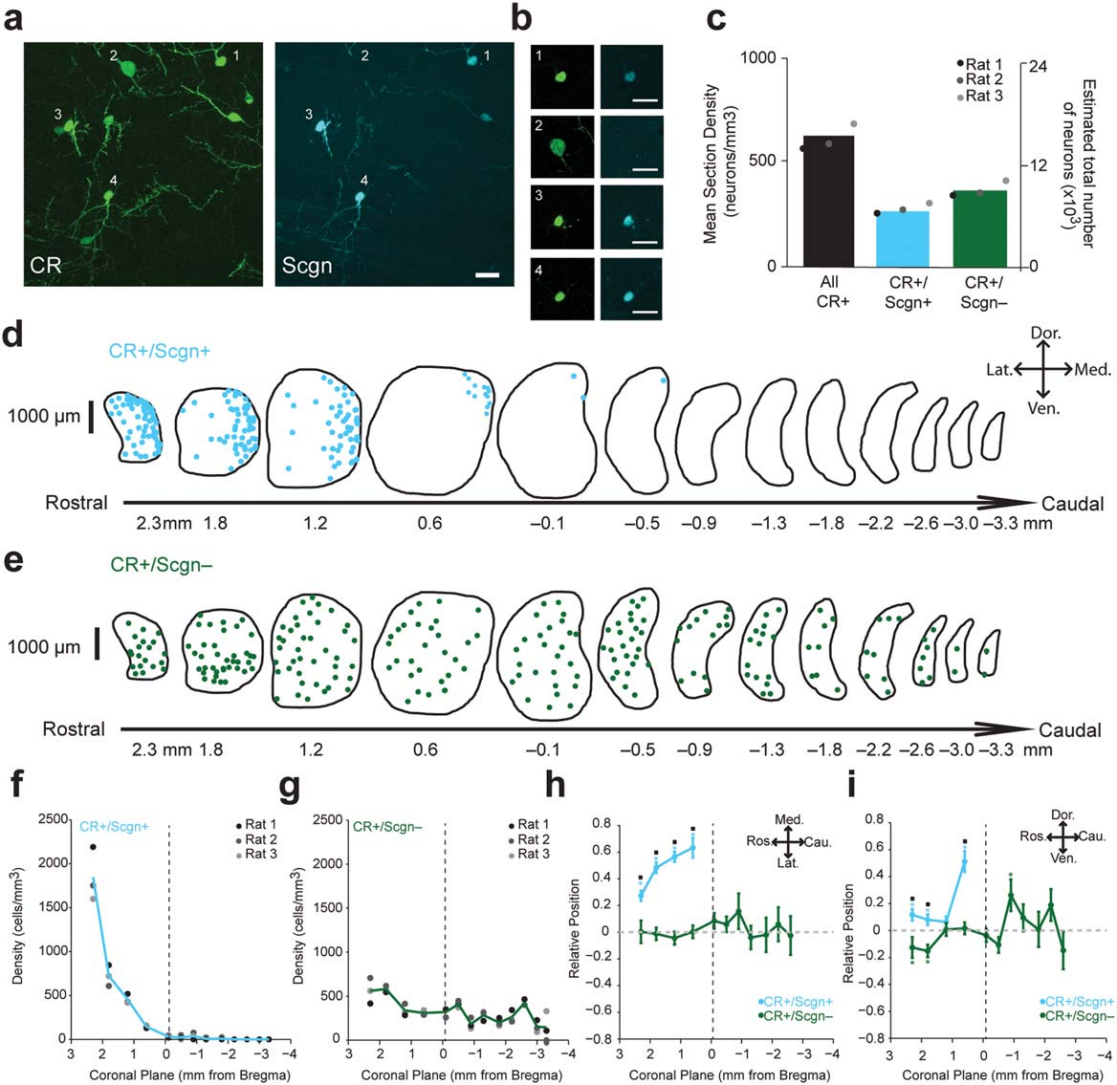
CR+/Scgn+ and CR+/Scgn– interneurons show different topographical distributions in the rat dorsal striatum. (a) Immunofluorescence signals of CR and Scgn in a rostral part of the rat dorsal striatum. Example CR+ interneurons that co‐express Scgn (cells 1, 3 and 4) and a CR+ interneuron that does not co‐express Scgn (cell 2). (b) Single‐plane confocal micrographs of the numbered neurons in panel (a). Note the smaller size, and stronger immunofluorescent signals for CR, exhibited by the Scgn‐expressing interneurons. Scale bars (a, b) = 20 µm. (c) The mean section density and estimated total number of all CR+, CR+/Scgn+ and CR+/Scgn− interneurons across all 13 coronal sections of dorsal striatum. Note that CR+/Scgn+ interneurons make up ∼40% of all CR+ interneurons in the dorsal striatum of the rat. (d, e) Distributions of CR+/Scgn+ interneurons (d) and CR+/Scgn− interneurons (e) across 13 coronal planes encompassing the rostro‐caudal axis of the dorsal striatum in a single rat, with each dot representing a single neuron in the 10 µm‐thick optical disector. (f, g) Densities of CR+/Scgn+ neurons (f) and CR+/Scgn− neurons (g) along the rostro‐caudal axis of rat striatum. Note the decrease in density of CR+/Scgn+ neurons when traversing from rostral to caudal striatum. (h, i) Medio‐lateral (h) and dorso‐ventral (i) distributions of CR+/Scgn+ interneurons (cyan line) and CR+/Scgn− interneurons (green line) along 13 coronal planes. In coronal planes where less than 5 neurons were counted per animal, no relative topographical value is given. The presence of asterisks (*) indicate a distribution that is significantly biased in one direction along the specified axis (Wilcoxon Signed Rank test). Black squares (▪) indicate a significant difference in the distribution of the two populations along the specified axis within a given coronal plane (Wilcoxon Rank Sum test). All significant values are indicated at *p* < .05. Data are means of the position of all neurons counted ± *SEMs*. (f–i) vertical dotted line on each plot shows the position of the anterior commissure

In the adult rat, the CBP secretagogin (Scgn) is expressed in a proportion of striatal interneurons (Garas et al., 2016; Kosaka et al., 2017; Mulder et al., 2009). We observed that some, but not all, CR+ interneurons co‐express Scgn (Figure 1a, b). In the CR+/Scgn+ neuron population, the expression of these CPBs was relatively intense, such that immunoreactivity for both markers could often be detected in proximal dendrites as well as in somata (Figure 1a). Previous descriptions of CR+ interneurons in the rat (Bennett & Bolam, 1993; Rymar et al., 2004) and mouse (Petryszyn et al., 2014) have noted a strong positional bias of these cells toward the rostral and medial borders of the dorsal striatum. Unbiased stereological cell‐counting revealed that, first, CR+/Scgn+ interneurons (total neurons counted = 462; rats = 3), represent approximately 40% of the total density of CR+ interneurons in the rat dorsal striatum (Figure 1c), and secondly, CR+/Scgn+ interneurons are mostly located in the rostral aspects of striatum (Figure 1d, f). This CR+/Scgn+ interneuron population is therefore unique among currently known types of striatal interneurons in that they are highly concentrated in the rostral (“pre‐commissural”) striatum and virtually absent in striatal planes caudal to the anterior commissure (Figure 1d, f). Analysis of the positioning of CR+/Scgn+ interneurons also revealed a strong medial bias (Figure 1h) and dorsal bias (Figure 1i). In contrast, those CR+ interneurons that did not co‐express Scgn (CR+/Scgn−; total neurons counted = 1031; rats = 3) were homogenously distributed throughout the rostro‐caudal axis (Figure 1e, g), medio‐lateral axis (Figure 1e, h) and dorso‐ventral axis (Figure 1e, i) of the rat striatum. In summary, the selective co‐expression of Scgn can be used to distinguish two topographically distinct populations of CR+ interneurons in the dorsal striatum of the rat.

### 3.2 The selective expression of Scgn is correlated with differences in the somatic diameters of CR+ interneurons in the rat dorsal striatum

**Figure 2:**
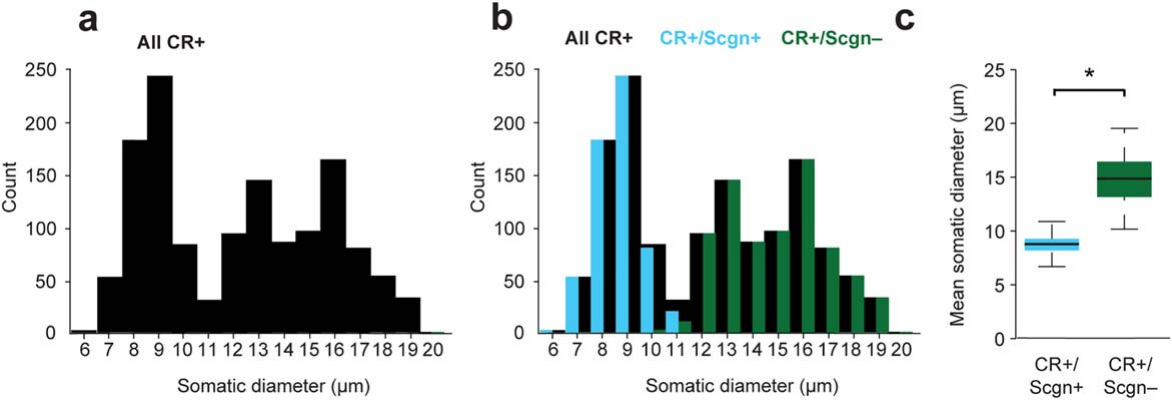
CR+/Scgn+ interneurons can be distinguished from CR+/Scgn− interneurons on the basis of their mean somatic diameters. (a) Frequency histogram showing the trimodal distribution of somatic diameters for CR+ interneurons in the dorsal striatum of three rats. (b) Frequency histogram of the same population as in (a), but now showing the distribution of somatic diameters of CR+/Scgn+ interneurons (cyan bars) and CR+/Scgn− interneurons (green bars). (c) CR+/Scgn+ interneurons were found to possess significantly smaller‐sized somata compared to CR+/Scgn− interneurons (Mann‐Whitney *U* test, *p* = 1.68 × 10^−189^). Data in plot (c) are the medians, the interquartile ranges (box), and extremes of the range (whiskers show the lowest and highest points within 1.5× the interquartile range, approximately 99% of the data for a normal distribution)

In accordance with previous studies of the rat dorsal striatum (Rymar et al., 2004), the somatic diameters of all CR+ interneurons varied from 6.2 to 19.6 µm in their longest axes (Figure 2a). A frequency histogram of somata sizes revealed a trimodal distribution, suggesting distinct groups (Figure 2a). Given previous reports that CR+ interneurons located in the rostral striatum of rodents tend to have relatively small somatic diameters (Petryszyn et al., 2014), and our finding that CR+/Scgn+ interneurons are rostrally biased in their distribution (Figure 1f), we measured the mean somatic diameter of CR+/Scgn+ interneurons and compared them with the diameters of CR+/Scgn− interneurons in the rat (Figure 2b). The CR+/Scgn+ interneurons accounted for the vast majority of CR+ interneurons with somata in the range of 7–11 µm, and did not exceed 12 µm in diameter (Figure 2b). In line with these results, the mean somatic diameter of CR+/Scgn+ interneurons was significantly smaller (8.8 ± 0.9 µm, range = 6.2–12.0 µm, *n* = 506) than that of CR+/Scgn− interneurons (15.0 ± 1.0 µm, range = 10.2–19.6 µm, *n* = 668, Mann Whitney *U* test, *p* = 1.68 × 10^−189^; Figure 2c).

### 3.3 Small‐sized CR+/Scgn+ interneurons also express the newborn neuron marker SP8 in the rat dorsal striatum

**Figure 3:**
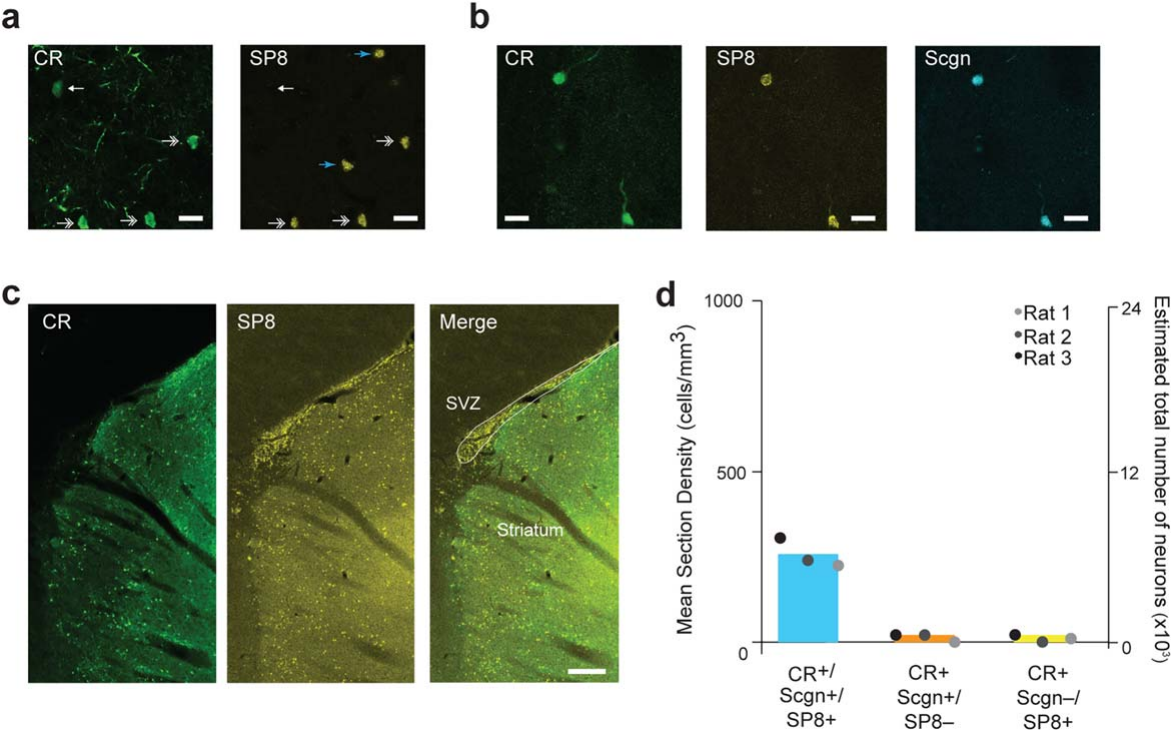
CR+/Scgn+ interneurons can also be identified by their expression of SP8. (a) Immunofluorescence signals of CR and SP8 in the rat dorsal striatum, showing CR+ interneurons that do (double arrows) and do not (white arrow) co‐express SP8. Some SP8‐expressing cells do not co‐express CR (blue arrows). (b) Neurons co‐expressing CR and SP8 also express Scgn. (c) Expression of CR and SP8 in a parasagittal section of the rat rostro‐medial striatum and subventricular zone (SVZ, outlined in merge panel). (d) Mean section densities and numbers of CR+/Scgn+/SP8+ interneurons, CR+/Scgn+/SP8− interneurons and CR+/Scgn−/SP8+ interneurons in the dorsal striatum. SP8 was rarely expressed in CR+ interneurons that did not co‐express Scgn. Scale bars: (a–d) 20 µm, (c) 200 µm

It has been previously reported that some CR+ interneurons in both rodents and primates are newly generated throughout adulthood from precursors located in the subventricular zone (Dayer, Cleaver, Abouantoun, & Cameron, 2005; Inta, Cameron, & Gass, 2015), which lies along the rostromedial borders the striatum. We therefore assessed the co‐expression of the transcription factor specificity protein 8 (SP8), a marker of newly generated neurons (Chung, Medina‐Ruiz, & Harland, 2014; Jiang, Zhang, You, & Liu, 2013), by CR+/Scgn+ and CR+/Scgn− interneurons in the rat dorsal striatum (Figure 3). Interestingly, while only some SP8+ striatal neurons were found to co‐express CR (Figure 3a), nearly all CR+/Scgn+ interneurons co‐expressed SP8 (99.7 ± 0.7%, *n* = 3 rats; Figure 3b, d). Importantly, CR+/Scgn− interneurons very rarely co‐expressed SP8 (Figure 3d). This suggests that much of population of the rostrally biased, small‐sized CR+/Scgn+ interneurons could be new born in adult striatum.

### 3.4 Larger‐sized CR+/Scgn− interneurons express the transcription factor Lhx7 in the rat dorsal striatum

**Figure 4:**
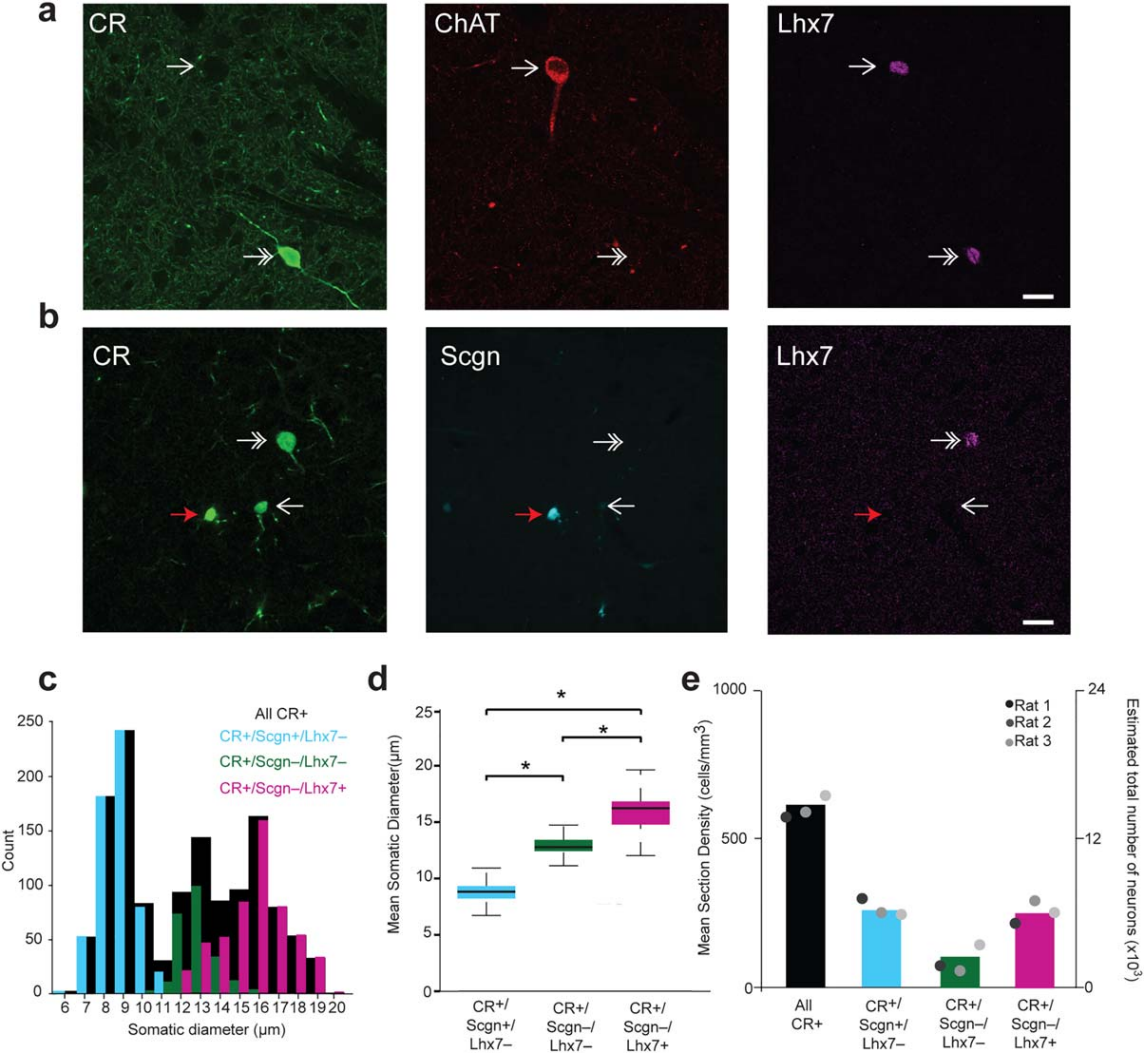
The selective expression of Lhx7 distinguishes some CR+/Scgn− interneurons and is correlated with differences in mean somatic diameter. (a, b) Immunofluorescence signals of CR, Scgn, Lhx7 and ChAT in the rat dorsal striatum. (a) Lhx7 is expressed in all ChAT+ interneurons (arrow) and some CR+ interneurons (double arrow). (b) CR+/Lhx7+ interneurons do not express Scgn (double arrow). Some CR+ neurons do not express Lhx7 or Scgn (white arrow) while others express Scgn, but not Lhx7 (red arrow). (c) Frequency histogram showing the distribution of somatic diameters of all CR+ interneurons (black bars), CR+/Scgn+/Lhx7− interneurons (cyan bars), CR+/Scgn−/Lhx7− interneurons (green bars) and CR+/Scgn−/Lhx7+ interneurons (magenta bars). Note that the somatic diameters of CR+/Scgn−/Lhx7− interneurons and CR+/Scgn−/Lhx7+ interneurons partially overlap and, therefore, somatic size cannot always be used to unambiguously distinguish between these cell populations. (d) Mean somatic diameters of immunochemically identified subpopulations of CR‐expressing interneurons. The presence of an asterisk indicates a level of significant difference as measured by a Kruskal–Wallis ANOVA followed by post hoc Dunn tests. (e) Mean section densities and estimated total numbers of interneurons with combinatorial expression of CR, Scgn and/or Lhx7. Of all CR+ interneurons in rat striatum, 40% were CR+/Scgn+/Lhx7−, 40% were CR+/Scgn−/Lhx7+ and the remainder CR+/Scgn−/Lhx7−. Scale bars: (a, b) 20µm. Data in plot (d) are the medians, the interquartile ranges (box), and extremes of the range (whiskers show the lowest and highest points within 1.5× the interquartile range, approximately 99% of the data for a normal distribution)

Having shown that small‐sized CR+ interneurons generally co‐express both Scgn and SP8, we further defined the molecular profile of the larger‐sized CR+/Scgn− interneurons. While many larger‐sized CR+ interneurons in the primate striatum co‐express ChAT (Petryszyn et al., 2014), in line with previous descriptions (Rymar et al., 2004), we could not find any examples of CR+/ChAT+ neurons in the rat striatum (Figure 4a). In the mouse striatum, expression of the transcription factor LIM homeobox protein 7 (Lhx7), also known as Lhx8 or L3 (Fragkouli, van Wijk, Lopes, Kessaris, & Pachnis, 2009; Zhao et al., 2003), is important in specifying the development (fate) of cholinergic interneurons (Fragkouli et al., 2009). When immunoreactivity for Lhx7, CR, Scgn, and ChAT was assessed, it was evident that Lhx7 is expressed by all ChAT+ interneurons, but also by some CR+/Scgn− neurons in rat dorsal striatum (Figure 4a, b). Importantly, the small‐sized CR+/Scgn+ interneurons did not express Lhx7 (Figure 4b). In order to investigate whether CR+/Scgn−/Lhx7+ and CR+/Scgn−/Lhx7− interneurons represented two distinct populations, the somatic diameters of these two molecularly distinct sets of CR+ interneurons were measured and compared with each other as well as with the somatic diameters of CR+/Scgn+ interneurons (Figure 4c). The somatic sizes of the three, molecularly defined CR+ interneurons subtypes were significantly different (Kruskal‐Wallis ANOVA, *p* = 1.3554 × 10^−209^). The somatic diameter of CR+/Scgn−/Lhx7+ interneurons (15.9 ± 1.7 µm, range = 12.0–19.6 µm, *n* = 460; Figure 4d) was significantly larger than that of CR+/Scgn−/Lhx7− interneurons (12.9 ± 0.99 µm, range = 10.2–16.2 µm, *n* = 208; post‐hoc Dunn test; Figure 4d). As expected, the somatic diameters of CR+/Scgn+/Lhx7− interneurons were also on average smaller than those of CR+/Scgn−/Lhx7− interneurons (post‐hoc Dunn test; Figure 4d). Taken together, these results suggest that the CR+/Scgn− interneurons in rat dorsal striatum can be further subdivided into a medium‐sized Lhx7− neurons and relatively large‐sized Lhx7+ neurons.

**Figure 5:**
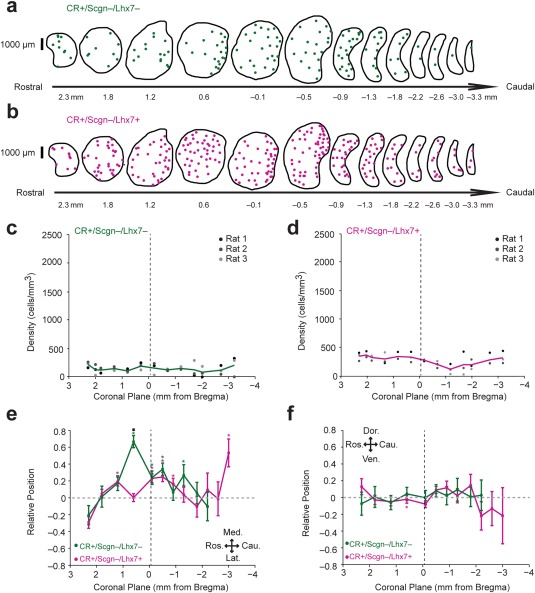
The topographical distributions of CR+/Scgn−/Lhx7− and CR+/Scgn−/Lhx7+ interneurons across the dorsal striatum of rat. (a, b) Distributions of CR+/Scgn−/Lhx7− interneurons (a) and CR+/Scgn−/Lhx7+ interneurons (b) across 13 coronal planes encompassing the rostro‐caudal axis of the dorsal striatum in a single rat, with each dot representing a single neuron. (c, d) Densities of CR+/Scgn−/Lhx7− interneurons (c) and CR+/Scgn−/Lhx7+ interneurons (d) along the rostro‐caudal striatal axis of rats. Note that both types of neurons are relatively consistent in their distribution from the rostral to the caudal pole of the striatum. (e, f) Medio‐lateral (e) and dorso‐ventral (f) distributions of CR+/Scgn−/Lhx7− interneurons (green line) and CR+/Scgn−/Lhx7+ interneurons (magenta line) along 13 coronal planes. In coronal planes where less than 5 neurons were counted per animal, no relative topographical value is given. The presence of asterisks (*) indicate a distribution that is significantly biased in one direction along the specified axis (Wilcoxon Signed Rank test). Black squares (▪; e–f) indicate a significant difference in the distribution of the two populations along the specified axis within a given coronal plane (Wilcoxon Rank Sum test). All significant values are indicated at *p* < .05. Data are means of the position of all neurons counted ± *SEMs*. (c–f) Vertical dotted line on each plot shows the position of the anterior commissure

Stereological counts demonstrated that CR+/Scgn−/Lhx7+ interneurons (total neurons counted = 695; rats = 3) represent nearly 40% and CR+/Scgn−/Lhx7− interneurons (total neurons counted = 336; rats = 3) represent 20% of the total density of the CR+ interneuron population in the rat dorsal striatum (Figure 4e). In agreement with previous results (Rymar et al., 2004), the total number of CR+ neurons in the rat dorsal striatum was estimated to be 13535 ± 392 cells. Unlike the rostrally biased distribution of CR+/Scgn+/Lhx7− interneurons within the rat dorsal striatum (Figure 1f), the mean densities of both CR+/Scgn−/Lhx7+ interneurons and CR+/Scgn−/Lhx7− interneurons were consistent across all striatal rostro‐caudal planes (Figure 5a–d). In those planes surrounding Bregma, CR+/Scgn−/Lhx7− interneurons, and (to a lesser degree) CR+/Scgn−/Lhx7+ interneurons, were medially biased in their distribution, but neither population was consistently biased along the dorso‐ventral axis (Figure 5e, f).

**Figure 6:**
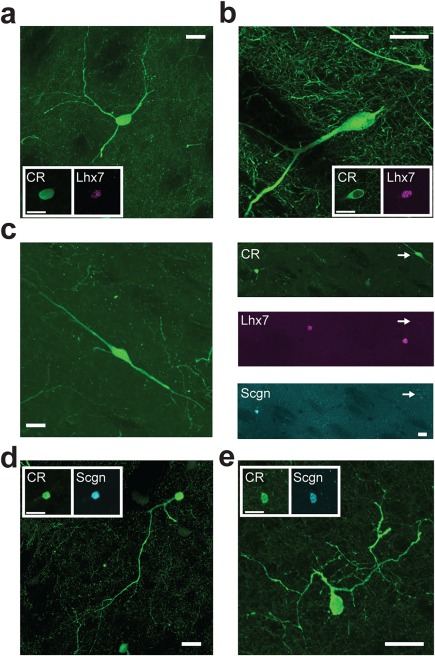
The selective expression of Scgn or Lhx7 identifies morphologically distinct “types” of CR‐expressing interneurons in rat striatum. (a–e) Confocal micrographs of the rat dorsal striatum showing the different morphological characteristics of CR‐expressing interneurons. (a, b) CR+/Lhx7+ (“Type 1”) interneurons had relatively large somata with 3 or more relatively thick primary dendrites. (c) CR+/Scgn−/Lhx7− (“Type 2”) interneurons (see arrows in panels on right) had medium‐sized somata and often had bipolar dendrites. (d, e) CR+/Scgn+ (“Type 3”) interneurons had small somata with tortuous dendrites that appeared to have some spine‐like appendages and “filopodia”. For (a–e), large panels show maximum intensity projections taken across multiple optical planes (z‐stacks) containing the extent of the labeled neuron's somatodendritic structure, while the boxed insets are single‐plane images of the somata of the same neurons. Scale bars (a–e) = 20 µm

We hypothesized that these groupings of CR+ interneurons on the basis of their molecular identity and somatic size would correspond to previous qualitative observations of heterogeneity in somatodendritic structure in the CR+ interneuron population (Petryszyn et al., 2014; Tepper et al., 2010). Confocal imaging of CR+ interneurons in the rat striatum suggested that the three groups we describe above broadly correspond with the three morphologically defined cell “types” suggested by Tepper and colleagues (Tepper et al., 2010). In addition to having relatively large somata, CR+/Scgn−/Lhx7+ interneurons consistently had thick primary dendrites that branched infrequently (“Type 1”, Figure 6a, b). In contrast, the medium‐sized CR+/Scgn−/Lhx7− interneurons has thinner, bipolar dendrites (“Type 2”, Figure 6c). Finally, the small‐sized CR+/Scgn+/Lhx7− interneurons (“Type 3”) possessed highly tortuous dendrites that often bore spine‐like appendages and filopodia (“Type 3”, Figure 6d, e).

Overall, these findings suggest that CR+ interneurons in the rat dorsal striatum are likely composed of at least three distinct cell types that can be identified on the bases of their molecular expression profiles, somatodendritic structures, and topographical distributions.

### 3.5 Spontaneous activity of identified CR‐expressing interneurons in the rat dorsal striatum in vivo

**Figure 7:**
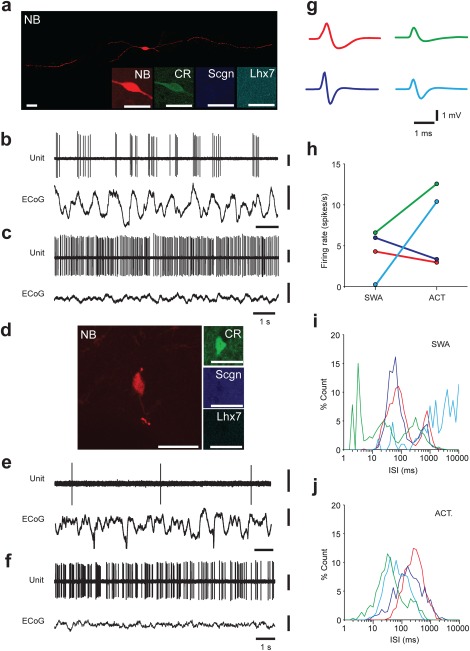
Spontaneous activity of identified CR+/Scgn−/Lhx7− striatal interneurons in the rat dorsal striatum. (a–f) Juxtacellularly labeled CR‐expressing interneurons (a, d), identified by their colocalization of fluorescent labeling for Neurobiotin (NB). Both interneurons tested negative for the expression of both Scgn and Lhx7. Scale bars = 20 µm. (b, e) Spontaneous action potential discharges (unit activity) of the same individual CR+ interneurons during cortical slow‐wave activity, as recorded in the frontal electrocorticogram (ECoG). (c, f) Firing of the same interneurons during spontaneous cortical activation. Scale bars for recordings represent 0.5 mV for the unit activity and 1 mV for the ECoG. (g) The mean action potential waveforms of the four CR+/Scgn−/Lhx7− interneurons recorded and identified in this study. (h) The mean firing rates of CR+/Scgn−/Lhx7− interneurons during both cortical slow‐wave activity and cortical activation. (i, j) ISI histograms of each recorded CR+ interneuron during cortical SWA (i) and cortical activation (j). (g–j) Note that the different colored lines represent one of the four CR+/Scgn−/Lhx7− interneurons

To our knowledge, the electrophysiological activity of identified CR+ interneurons in the dorsal striatum has yet to be recorded in vitro or in vivo. During more than 300 experiments over 6 years in which we performed extracellular recording and juxtacellular labeling of individual striatal neurons in anesthetized rats, we recorded four striatal interneurons that were post hoc identified as expressing CR. This small sample, in contrast with the relatively larger number of other interneuron types recorded across these experiments (Garas et al., 2016), is likely due to the difficulty of detecting such sparse interneurons in a “blinded” manner using glass electrodes in vivo. After confirming that these recorded interneurons expressed CR, their somata were further tested for expression of both Scgn and Lhx7; all four recorded interneurons were CR+/Scgn−/Lhx7− (“Type 2”, Figure 7a, d). During cortical SWA, the firing patterns of CR+ neurons were highly variable (Figure 7b, e). For example, some neurons fired “bursts” of spikes around the peaks of most cortical slow oscillations (Figure 7b) while others fired single spikes in a sporadic fashion (Figure 7e). In contrast, during spontaneous cortical activation, all of these CR+ interneurons displayed tonic firing (Figure 7c, f). The action potential waveforms of CR+/Scgn−/Lhx7− interneurons were also heterogeneous (Figure 7g), with some neurons displaying brief/fast (<1 ms) spikes while others had longer spike waveforms (>1.5 ms). The firing rates of CR+ interneurons were also highly variable during both cortical SWA and cortical activation (Figure 7h). Spontaneous changes in cortical activity from SWA to an activated state were accompanied by large increases in the firing rates of two interneurons, while the other two interneurons exhibited relatively small decreases in firing rate during similar state transitions (Figure 7h). During SWA, the ISI histograms of some interneurons were bimodal (Figure 7i; red and purple lines), with a first peak at 100 ms and a second peak at ∼1 s, which likely reflects their tendency to fire in time with the cortical slow oscillation (Figure 7b). During cortical activation, CR+ interneurons fired more regularly (Figure 7c, f), reflected in ISI histograms by a single peak (Figure 7j), which roughly correlated with the mean firing rate of that neuron (Figure 7h). Overall, the most consistent property of these CR+ interneurons was their sustained, relatively regular firing during the activated state.

### 3.6 Molecular and morphological subtypes of CR+ interneurons described in the rat are also present in the mouse dorsal striatum

**Figure 8:**
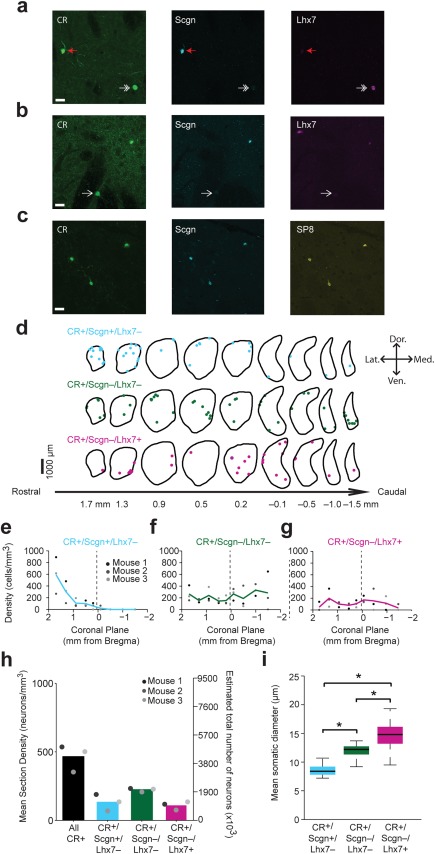
A combination of CR, Scgn, SP8 and Lhx7 immunohistochemistry identifies three populations of CR+ interneurons that differ in their molecular profiles, topographical distributions and mean somatic diameters in the mouse dorsal striatum. (a, b) Immunofluorescence signals of CR, Scgn, and Lhx7 in the mouse dorsal striatum. CR+ interneurons that co‐express Scgn do not express Lhx7 (a; red arrow). Similarly, CR+ interneurons that co‐express Lhx7 do not express Scgn (a; double arrow). Some CR+ interneurons do not co‐express either Scgn or Lhx7 (b; white arrow). (c) CR+ interneurons that co‐express Scgn also express SP8. Scale bars in (a–c) represent 20 µm. (d) Distributions of CR+/Scgn+/Lhx7−, CR+/Scgn−/Lhx7− and CR+/Scgn−/Lhx7+ neurons across 9 coronal planes encompassing the rostro‐caudal axis of the dorsal striatum in a single mouse, with each dot representing a single neuron in the 10 µm‐thick optical disector. (e–g) Densities of CR+/Scgn+/Lhx7– neurons (e), CR+/Scgn−/Lhx7− neurons (f) and CR+/Scgn−/Lhx7+ neurons (g) along the rostro‐caudal striatal axis. Note the decrease in density of CR+/Scgn+/Lhx7− neurons when traversing from rostral to caudal striatum, while neither CR+/Scgn−/Lhx7− neurons nor CR+/Scgn−/Lhx7+ neurons are biased toward either striatal pole. (h) The mean section densities and estimate of the total numbers of all CR+, CR+/Scgn+/Lhx7−, CR+/Scgn−/Lhx7− and CR+/Scgn−/Lhx7+ interneurons within the entirety of the mouse dorsal striatum. (i) Mean somatic diameter of immunochemically identified subpopulations of CR‐expressing interneuron. A single asterisk indicates a level indicates a significant as measured by a Kruskal‐Wallis ANOVA test followed by post‐hoc Dunn tests. Data in plot (i) are the medians, the interquartile ranges (box), and extremes of the range (whiskers show the lowest and highest points within 1.5× the interquartile range, approximately 99% of the data for a normal distribution). (e–g) Vertical dotted line on each plot shows the position of the anterior commissure

We next investigated the molecular and morphological properties of CR+ interneurons in the dorsal striatum of adult mice. In agreement with previous work, we saw no evidence of CR+ interneurons in mice co‐expressing ChAT (Petryszyn et al., 2014). Immunofluorescence labeling of CR, Scgn, and Lhx7 revealed the existence of CR+/Scgn+/Lhx7− interneurons (total neurons counted = 94; mice = 3), CR+/Scgn−/Lhx7− interneurons (total neurons counted = 167; mice = 3) and CR+/Scgn−/Lhx7+ interneurons (total neurons counted = 91; mice = 3) in the dorsal striatum of the mouse (Figure 8a, b). As in the rat, Lhx7 and Scgn were not co‐expressed together by CR+ interneurons (Figure 8a,b), and CR+/Scgn+/Lhx7− interneurons represented the only group of CR+ interneurons that also expressed SP8 (Figure 8c). Stereological cell counting across 9 coronal planes was performed in order to estimate the total numbers and spatial distributions of CR+ interneurons (Figure 8d). When the volume of the mouse dorsal striatum is taken into consideration, the total number of CR+ interneurons was estimated to be 4095 ± 195 cells. The number of counted neurons per striatal section in the mouse was often too low for meaningful statistical analysis of interneuron distribution, but topographical mapping nevertheless revealed that CR+/Scgn+/Lhx7− interneurons represented approximately one‐quarter of all CR+ interneurons by density, and were biased toward the rostral pole of the dorsal striatum (Figure 8e, h). In contrast, CR+/Scgn−/Lhx7− interneurons represented nearly half of all CR+ interneurons by density, and were distributed evenly throughout the striatum (Figure 8f, h). Additionally, CR+/Scgn−/Lhx7+ interneurons represented nearly one‐quarter of CR+ interneurons, and were not strongly biased in their distributions (Figure 8g, h). In accordance with previous estimates (Petryszyn et al., 2014), we report that the mean density of all CR+ interneurons in the dorsal striatum mouse is similar to that of the rat (Figure 8h).

**Figure 9:**
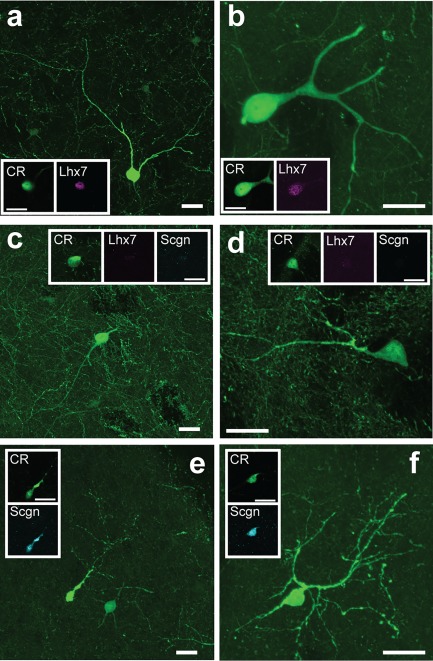
The selective expression of Scgn or Lhx7 identifies morphologically distinct “types” of CR‐expressing interneurons in mouse striatum. (a–f) Confocal micrographs of the mouse dorsal striatum showing the different morphological characteristics of CR‐expressing interneurons. (a, b) CR+/Lhx7+ (“Type 1”) interneurons had large somata with 3 or more relatively thick primary dendrites. (c, d) CR+/Scgn−/Lhx7− (“Type 2”) interneurons had medium‐sized somata and often had bipolar dendrites. (e, f) CR+/Scgn+/Lhx7− (“Type 3”) interneurons had small somata and tortuous dendrites. For (a–f), large panels show maximum intensity projections taken across multiple optical planes (z‐stacks) containing the extent of the labeled neuron's somatodendritic structure, while the boxed insets are single‐plane images of the somata of the same neurons. All scale bars are 20 µm

As in the rat, the mean somatic diameters of the three molecularly defined neuron types were significantly different (Kruskal‐Wallis ANOVA, *p* = 2.36 × 10^−19^). On average, the somata of CR+/Scgn+/Lhx7− interneurons (8.5 ± 0.9 µm, range = 7.2–10.7 µm, *n* = 45) were significantly smaller than those of CR+/Scgn−/Lhx7− interneurons (11.9 ± 1.4 µm, range = 8.5–15.7 µm, *n* = 49; post‐hoc Dunn test) and CR+/Scgn−/Lhx7+ interneurons (14.7 ± 2.3 µm, range = 9.5–19.3 µm, *n* = 39; post‐hoc Dunn test, Figure 8i). Moreover, the somata of CR+/Scgn−/Lhx7− interneurons were significantly smaller than those of CR+/Scgn−/Lhx7+ interneurons (post‐hoc Dunn test, Figure 8i). Thus, in the mouse, the distinct molecular profiles of three striatal CR+ interneuron subpopulations are reflected in the distinct sizes of their somata, and these distinctions are similar to those of CR+ interneurons in the rat (Figure 4d). Confocal imaging of the CR immunofluorescence signal in mouse revealed further structural similarities of these molecularly defined interneuron types across mice and rats. Thus, CR+/Scgn−/Lhx7+ interneurons were generally large‐sized, with thick primary dendrites that branched infrequently (Figure 9a, b), features which closely resemble the “Type 1” CR+ interneurons of the rat. Moreover, CR+/Scgn−/Lhx7− interneurons in the mouse had medium‐sized somata (Figure 9c, d), and were thus structurally similar to the “Type 2” CR+ interneurons in rats. The small‐sized CR+/Scgn+/Lhx7− interneurons were irregularly shaped with dendrites that branched frequently and appeared to possess spine‐like appendages (Figure 9e, f), similar to those of “Type 3” CR+ interneurons in rats. Taken together, these results largely suggest that CR+ interneurons in the mouse are analogous to those in the rat with respect to their molecular and structural properties.

### 3.7 A discrete population of CR+ interneurons also express Scgn in the primate caudate/putamen

Previous descriptions of CR+ interneurons in the primate striatum have described a rostrally biased distribution and three morphological subtypes (small‐, medium‐, and large‐sized) (Petryszyn et al., 2014; Petryszyn et al., 2017). Given our findings in rats and mice, we next aimed to investigate whether (a) co‐expression of Scgn in primate striatal CR+ interneurons defined a rostrally biased population, and (b) the combination of Scgn/SP8/Lhx7 could reliably separate the three reported morphological CR+ interneuron subtypes in primates. Regrettably, the anti‐SP8 and anti‐Lhx7 antibodies used in the rat and mouse failed to produce reliable and consistent results in the rhesus macaque tissue and thus only Scgn expression was pursued further.

**Figure 10:**
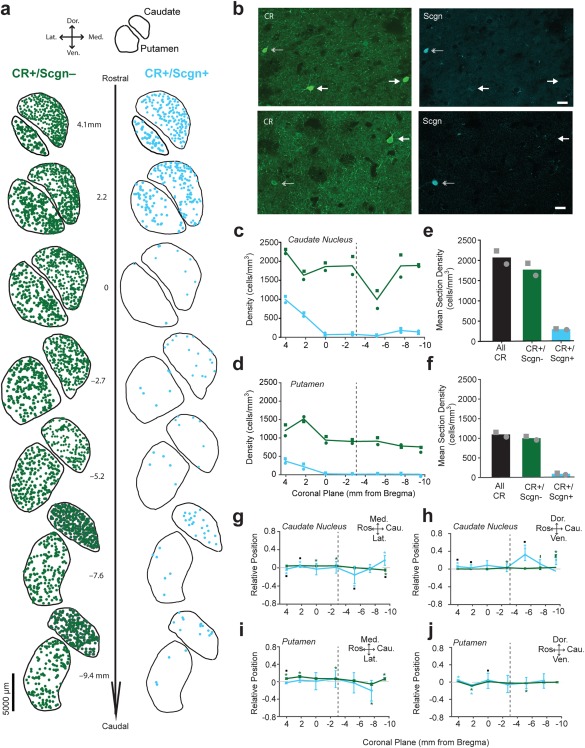
CR+/Scgn+ and CR+/Scgn− interneurons have different topographical bias in the caudate and putamen of the rhesus macaque. (a) Distributions of CR+/Scgn− interneurons (green; on left) and CR+/Scgn+ interneurons (cyan; on right) across 7 coronal planes of the caudate and putamen in a single monkey, with each dot representing a single neuron in the 10‐µm‐thick optical disector. (b) Confocal micrographs of the monkey striatum showing that some CR+ interneurons co‐expressed Scgn (double arrow) while some did not (arrows). Scale bars = 20 µm. (c, d) Densities of CR+/Scgn+ interneurons (cyan line) and CR+/Scgn− interneurons (green line) along the rostro‐caudal axis of the caudate (c) and putamen (d). Note that the CR+/Scgn+ cell population in the monkey increases in density toward the rostral planes of the caudate and the putamen. (e, f) Mean section densities of CR+ interneurons across the entirety of the caudate nucleus (e) and the putamen (f), including those subpopulations that co‐express Scgn (cyan bar) and do not express Scgn (green bar). In the monkey caudate‐putamen, CR+/Scgn+ interneurons represent nearly 10% of all CR‐expressing neurons. (g–j) Distributions of CR+/Scgn+ interneurons (cyan line) and CR+/Scgn− interneurons (green line) along the medio‐lateral (g, i) and dorso‐ventral (h, j) axes of the caudate nucleus (g, h) and putamen (i, j). The presence of asterisks (*) indicate a distribution that is significantly biased in one direction along the specified axis (Wilcoxon Signed Rank test). Black filled squares (▪; g–j) indicate a significant difference in the distribution of the two populations along the specified axis within a given coronal plane (Wilcoxon Rank Sum test). All significant values are indicated at *p* < .05. Data are means of the position of all neurons counted ± *SEMs*. (c–f) Square and circles show mean values for Monkey 1 and Monkey 2, respectively. (c, d, g–j) Vertical dotted line on each plot shows the position of the anterior commissure

Immunofluorescent detection of CR and Scgn in seven coronal sections containing the primate caudate and putamen revealed that a proportion of CR+ interneurons do co‐express Scgn (Figure 10a, b; total number of CR+/Scgn+ neurons counted = 719; monkeys = 2). Indeed, CR+/Scgn+ interneurons were almost entirely restricted to the head of the caudate nucleus and the pre‐commissural putamen (Figure 10a, c, d), which is reminiscent of the strong rostral bias in the positioning of these interneurons in the rat and mouse striatum (Figures 1d, f, 8d, e). In contrast, CR+/Scgn– interneurons (total neurons counted = 5305; monkeys = 2) were distributed throughout the rostro‐caudal extent of both the caudate nucleus and the putamen (Figure 10a, c, d), which is again reminiscent of the wide distribution of this cell population in rodent striatum (Figures 1 e, g, 8d, f, g). In agreement with previous studies (Deng, Shelby, & Reiner, 2010), the density of all CR+ interneurons was higher in the caudate nucleus (Figure 10c, e) than in the putamen (Figure 10d, f). Moreover, the mean section densities of CR+ interneurons in primate striatum (Figure 10e, f) were far greater than those in rodent striatum (Figures 1c, 4e, 8h). Of note, CR+/Scgn+ interneurons only accounted for ∼10% of all CR+ interneurons by density in the primate striatum (Figure 10e, f), compared to ∼40% in the rat (Figure 1c) and ∼25% in the mouse (Figure 8h). The mean somatic diameters of CR+/Scgn+ interneurons did not significantly differ from CR+/Scgn− interneurons in either the caudate or the putamen (data not shown). The distribution of CR+/Scgn+ neurons was not consistently biased in the medio‐lateral or dorso‐ventral axes in the primate (Figure 10g–j). Taken together, these data suggest that, while CR+/Scgn+ interneurons make up approximately 10% of all CR‐immunoreactive neurons across primate striatum, they account for up to 30% in the head of the caudate nucleus and the pre‐commissural putamen. The selective expression of Scgn therefore identifies a topographically biased subpopulation of CR+ interneurons in the primate striatum.

## 4 DISCUSSION

The results presented in this study provide the most comprehensive correlations to date between the molecular expression profiles, morphological properties and topographical distributions of CR+ interneurons in the dorsal striatum of the rat, mouse, and primate. We demonstrate that, in rodents, the expression of different combinations of Scgn, SP8, and Lhx7 correlate strongly with the topographic and structural properties of CR+ interneurons. Together, these findings suggest that there are at least three distinct classes of CR+ interneurons in the striatum of the rat and the mouse. Furthermore, the spatial distribution of CR+/Scgn+ interneurons in primates has striking similarities to that of rodents, suggesting that these properties are conserved to some extent throughout phylogeny. In addition, we provide the first electrophysiological recordings from a small number CR+ interneurons, which demonstrate that some striatal CR+ interneurons can be “tonically active” in vivo.

### 4.1 Secretagogin co‐expression delineates small‐sized rostrally biased CR+ interneurons that may be adult‐born

The most conspicuous subtype of CR+ interneurons in rodents have small somata and tortuous, spiny dendrites, and are found almost exclusively in the rostromedial striatum (“Type 3”). These CR+ interneurons have been described previously (Petryszyn et al., 2014; Revishchin, Okhotin, & Pavlova, 2010; Tepper et al., 2010; Wei et al., 2011) and are thought to be cells that migrate postnatally from the SVZ into the rostromedial striatum, where they mature into CR+ interneurons (Dayer et al., 2005; Inta et al., 2015; Luzzati, De Marchis, Fasolo, & Peretto, 2006). In rodents, this process is thought to occur throughout adulthood (Revishchin et al., 2010) and is accelerated in response to stroke (Wei et al., 2011). We provide further evidence that these small‐sized CR+ interneurons represent a distinct subpopulation, by demonstrating that they can be reliably identified based on their co‐expression of Scgn and SP8. Carbon‐14 dating suggests that CR+ interneurons also migrate from the SVZ into the rostromedial striatum throughout adulthood in humans (Ernst et al., 2014). However, another study, primarily using transcription factor expression, concluded that neuroblasts from the SVZ do not migrate into the striatum in humans and primates (Wang et al., 2014). Notably, we found that there was no difference in somatic size between the CR+/Scgn+ interneurons (putative new‐born, based on rodent tissue) and CR+/Scgn− interneurons in primates. Thus, despite the similar rostro‐caudal bias of CR+/Scgn+ in rodents and primates, this combination of markers may not be indicative of putative new‐born CR+ interneurons in monkeys. Interestingly, the proportional densities of CR+ interneurons expressing Scgn varied considerably across species. Given that CR+/Scgn+ interneurons in rodents have highly similar molecular and structural properties, this could indicate a more prominent role for this cell type in governing striatal function in the rat as compared to mouse. The relatively small proportion of CR+/Scgn+ interneurons in primates suggests that the greater abundance of CR+ interneurons as a whole (Petryszyn et al., 2014; Petryszyn, Di Paolo, Parent, & Parent, 2016) is driven to a large extent by a selective proliferation of CR+/Scgn− neurons. A greater understanding of other functional properties of the different classes of CR+ interneuron is needed to further interpret the relevance of these species differences.

The biased rostromedial distribution of these CR+/Scgn+ interneurons in rodent striatum is particularly striking. The broad function of neurons in the striatum generally corresponds to the type of cortical input to a given territory (Hintiryan et al., 2016; Hunnicutt et al., 2016). The rodent rostromedial striatum receives cortical projections primarily from associative cortices in the rat (Hoover & Vertes, 2011; Mailly, Aliane, Groenewegen, Haber, & Deniau, 2013). However, to our knowledge, there is no information about the extent to which these putative newborn neurons are “functionally embedded” into the striatal microcircuit. Thus, in this case, proximity to the SVZ could be an equally or more important factor underlying the high density of this cell population in rostromedial areas of striatum. Interestingly, we found that CR+/Scgn+ interneurons in the primate striatum were also considerably more abundant in rostral areas, particularly the head of the caudate nucleus, that receive input from associative cortices (Yeterian & Pandya, 1993, 1998). As we could not test whether these monkey CR+/Scgn+ interneurons co‐expressed SP8, further work will be needed to establish whether these interneurons are also putative adult‐born cells or represent a topographically specific population with a function related to those associative cortical areas in primates. In either case, and as recently described for parvalbumin (PV)‐expressing striatal interneurons (Garas et al., 2016), Scgn appears to delineate a specialized subpopulation of CR+ interneurons in rodents and primates.

### 4.2 Medium‐sized CR+ interneurons do not express Lhx7 or secretagogin

Early qualitative descriptions of CR+ interneurons in the rodent dorsal striatum suggested they were “medium‐sized” (Bennett & Bolam, 1993; Jacobowitz & Winsky, 1991), an observation later extended to monkeys (Parent et al., 1996) and humans (Cicchetti et al., 1998). Our use of Scgn and Lhx7 immunoreactivity, as well as an in‐depth investigation of somatic size, revealed that medium‐sized CR+ interneurons (as defined by their somatic diameter and their lack of Scgn or Lhx7 expression) make up 20% of all CR+ interneurons in the rat and nearly 50% of all CR+ interneurons in the mouse. These “Type 2” medium‐sized CR+/Scgn−/Lhx7− interneurons were preferentially distributed in the medial striatum in rats. In the primate striatum, we found that CR+/Scgn− interneurons (irrespective of Lhx7, which was not tested) did not demonstrate any strong or consistent distributional bias within either the caudate or the putamen. This is in agreement with many studies of interneuron distribution in the primate striatum, where any preference for localizing to one particular region was not detected (Deng et al., 2010; Petryszyn et al., 2014, 2016). The rostral distribution of CR+/Scgn+ interneurons presented here does, however, provide evidence that striatal interneuron populations in the primate can be heterogeneously distributed, in line with the recent demonstration of biased distributions for different types of PV+ interneurons in primates (Garas et al 2016). Future molecular phenotyping of CR+/Scgn+ and CR+/Scgn− interneurons could reveal additional subtypes with topographical bias in the primate.

### 4.3 Medium‐sized CR+ interneurons are “tonically active” during cortical activation in the rat

The functional roles performed by GABAergic CR+ interneurons within the striatal microcircuit are unclear, which is partly due to the lack of data regarding their local synaptic connectivity and electrophysiological properties (Tepper et al., 2010). To our knowledge, we herein provide the first definition of the in vivo electrophysiological properties of identified CR+ interneurons. By chance, all of our single‐cell recordings were made from medium‐sized, Type 2 CR+ interneurons (CR+/Scgn−/Lhx7−). Our recordings suggest that CR+/Scgn−/Lhx7− interneurons can be “fast spiking” (i.e., have a spike duration of around 1 ms) and have generally heterogeneous firing rates and patterns, particularly during cortical slow‐wave activity. The most consistent feature of these interneurons was there near continuous, regular firing at 3–12 spikes/s during cortical activation. These firing properties resemble those of cholinergic interneurons and some PV‐expressing interneurons (Sharott et al. 2012; Doig et al. 2014; Garas et al, 2016), but not of SPNs or nitric oxide synthase‐expressing interneurons (Sharott et al., 2012; Sharott et al., 2017), as also recorded in this brain state in anesthetized rats. If these firing properties of CR+/Scgn−/Lhx7− interneurons are maintained in awake, behaving animals, this cell type might be confused (erroneously grouped) with putative cholinergic interneurons, which are often assigned this identity on the basis of “tonically active” firing patterns (Goldberg & Reynolds, 2011). In rodents, the sparsity of these CR+ interneurons makes it likely that they would constitute only a small number of recorded neurons in behaving animals. In primates, however, where CR+ interneurons are far more numerous, extracellular recordings of tonically active neurons could encompass both cholinergic interneurons and a significant number of CR+ interneurons. It should be noted that, if the waveform of CR+ interneurons were to differ from the long, triphasic action potential of putative cholinergic interneurons, careful examination of the action potential waveform could partly resolve this ambiguity (Adler, Katabi, Finkes, Prut, & Bergman, 2013). Given the high density of CR+ neurons in the primate striatum and the importance of primate data in elucidating the role of different striatal interneuron types in relation to behavior, our data suggests that this issue at least warrants further exploration in these primate data sets.

### 4.4 Lhx7 expression in the large‐sized CR+ interneurons of the rodent may link them to the large CR+/ChAT+ expressing interneurons of the primate

In the striatum of squirrel monkeys (Parent et al., 1996) and humans (Cicchetti et al., 1998), many large‐sized (>20 µm) CR+ interneurons co‐express ChAT and are highly analogous in somatodendritic structure to the large striatal cholinergic interneurons that do not co‐express CR (Cicchetti et al., 2000; Parent et al., 1996). Although ChAT and CR are not co‐localized in interneurons of the rodent striatum, relatively large (>15 µm) CR+ interneurons have been described in the striatum of both mice (Petryszyn et al., 2014) and rats (Rymar et al., 2004). Here, we show that these larger CR+ interneurons selectively express the transcription factor Lhx7, which is required for the development of cholinergic interneurons from embryonic medial ganglionic eminence (MGE)‐derived cell progenitors (Fragkouli et al., 2009). This finding suggests that CR+ interneurons co‐expressing Lhx7 could constitute a separate subtype that is related in lineage to ChAT+ interneurons. If so, the relatively large‐sized CR+/Scgn−/Lhx7+ interneurons of the rodent could be related to the large CR+/ChAT+ interneurons of the primate. However, although the CR+/Scgn−/Lhx7+ interneurons are the largest of CR+ interneurons, they are smaller than ChAT+ interneurons in rodents and primates (Petryszyn et al., 2016; Petryszyn et al., 2017). This observation supports the alternative premise that ChAT+/CR− and ChAT+/CR+ interneurons in primates are more strongly related to ChAT+ interneurons in rodents, with CR+/Scgn−/Lhx7+ interneurons comprising an entirely distinct cell type. Further molecular characterization of striatal interneurons across the life courses of rodents and primates will be needed to resolve this issue. Whatever the case, the expression of Lhx7 by large “Type 1” CR+ interneurons in the adult rat is by itself significant, as it suggests that these neurons are also derived from the MGE, whereas CR+/Scgn+/Lhx7− interneurons expressing SP8 are likely to be derived from the SVZ or embryonic caudal ganglionic eminence (Inta et al., 2015). This implied difference in developmental linage, as revealed by the selective expression of Scgn or Lhx7, provides further evidence that the molecular characterization described here identifies a minimum of three types of striatal CR+ interneuron. In mice, the density of CR+/Scgn−/Lhx7+ interneurons was around half that of CR+/Scgn−/Lhx7− interneurons, whereas these proportions were approximately reversed in rats. This could reflect further differences between the striatal microcircuits of mice and rats, in addition to those we have recently described for parvalbumin‐expressing interneurons (Garas et al., 2016).

The present study provides a novel correlative approach to identify different populations of morphologically, topographically, and molecularly distinct CR+ interneurons in the striatum of both mice and rats. One of these populations, namely rostrally biased CR+/Scgn+ interneurons, was partially conserved between the rodent and primate. Our molecular characterization of striatal CR+ interneurons using Scgn and Lhx7 may also provide a useful starting point for the design of cell‐type‐selective manipulations that can be used to disentangle the specialized contributions of different populations of CR+ interneurons to activity dynamics within the striatal microcircuit.

## CONFLICT OF INTEREST

The authors declare no competing financial interests.
